# Microsatellite instability as prognostic marker in bladder tumors: a clinical significance

**DOI:** 10.1186/1471-2490-5-2

**Published:** 2005-01-12

**Authors:** Minal Vaish, Anil Mandhani, RD Mittal, Balraj Mittal

**Affiliations:** 1 Departments of Genetics, Sanjay Gandhi Post Graduate Institute of Medical sciences, RaiBariely Road, Lucknow- 226014, India; 2 Department of Urology and Renal Sciences, Sanjay Gandhi Post Graduate Institute of Medical sciences, RaiBariely Road, Lucknow- 226014, India; 3 Malaria lab, International Centre for Genetic Engineering & Biotechnology, Aruna Asaf Ali Marg, New Delhi-110067

## Abstract

**Background:**

Carcinoma of urinary bladder is one of the leading causes of death in India. Successful treatment of bladder cancer depends on the early detection & specific diagnostic approaches. In the present study, microsatellite instability (MSI) has been evaluated as a prognostic marker in patients with superficial urinary bladder cancer in lower urinary tract for determining risk of recurrence.

**Methods:**

A total of 44 patients with bladder tumors diagnosed with Transitional Cell Carcinomas [TCC] from lower urinary tract were selected for the study. Tumors were staged and graded according to AJCC-UICC (1997) classification and patients were followed with cystoscopy as per the protocol. Polymerase chain reaction (PCR) was done to amplify microsatellite sequences at mononucleotide BAT – 26, BAT – 40, TGFβ RII, IGFIIR, hMSH3, BAX and dinucleotide D2S123, D9S283, D9S1851 and D18S58 loci in blood (control) and tumor DNA. PCR products were separated on 8% denaturing polyacrylamide gel and visualized by autoradiography.

**Results:**

MSI was observed in 72.7% of tumors at BAT – 26, BAT – 40, D2S123, D9S283, D9S1851 and D18S58 loci. Good association of MSI was seen with tumor stage and grade. MSI – High (instability at > 30% of loci) was frequently observed in high stage (40.6%) and high grade (59.4%) tumors. Of 24 tumors of Ta-T1 stage with different grades, 11 (9/18 high grade and 2/6 low grade tumors) recurred in the mean duration of 36 months. MSI positivity was significantly high in patients who had one or more recurrences (p = 0.02 for high grade and 0.04 for low grade tumors).

**Conclusions:**

MSI may be an independent prognostic marker for assessing risk of recurrence in superficial tumors irrespective of the grade. Further studies on progression would help in stratifying the patients of T1G3 for early cystectomy vs bladder preservation protocol.

## Background

Amongst the genitourinary cancer, carcinoma of the urinary bladder is one of the leading causes of death in Indian population. Transitional cell carcinoma (TCC) is the commonest histopathological variant where stage and grade are the two important prognostic factors to know the clinical behavior of these tumors. Superficial tumors with different grades behave differently e.g. tumors with high grade recur early and progress to invasive bladder cancer very soon. This behavior of same stage of the tumor but with varied grades is attributed to genetic alterations.

Bladder cancer manifesting from superficial to aggressive muscle invasive tumors undergoes a sequence of genetic alterations. Primary chromosomal aberrations are associated with tumor development while secondary chromosomal abnormalities lead to progression to a more advanced stage. A frequent loss of heterozygosity (LOH) on chromosomes 4, 5, 8, 9, 11 and 17 is considered a major event in the carcinogenesis of bladder cancer [1,2].

Defects in mismatch repair (MMR) genes result in replication errors and genetic instability. Faulty mismatch repair, generally observed as somatic variation in size of microsatellites (short tandem repeat sequences in genome) is referred as microsatellite instability (MSI) [3]. MSI and LOH in bladder cancer have been reported by several investigators [4,5].

A successful treatment of bladder cancer depends on early detection and more specific diagnostic approaches. Preneoplastic changes of the bladder epithelium or superficial tumors as an early event precede invasive bladder carcinomas. Though the higher grade and stage portends a worst prognosis, superficial tumors of same stage and grade have different outcome in different patients. Due to limited utility of these prognosticators in patients with superficial bladder tumor, there is a need to analyze new molecular parameters in predicting the prognosis and risk of recurrence. The following study is based on MSI analysis in tumor tissues to evaluate its utility as a marker for early detection of recurrent bladder carcinomas from lower urinary tract and thus help in deciding treatment modalities.

## Methods

### Patient selection

Total of 44 patients with male & female ratio of (42:2) of TCC with a mean age of 62 years were included for the study after the approval from ethical committee. All the patients selected for the study were not having any familial cancer syndrome or had previous history of cancer to the best of our knowledge.

All the tumors were resected transurethrally from the lower urinary tract. Part of superficial tissue specimen obtained after transurethral resection of bladder tumor (TURBT) was collected immediately in liquid nitrogen. Matched control sample (5 ml of peripheral blood) from all patients was collected in 200 μl of 0.5 M EDTA. The tumor stage and grade was assigned according to the TNM classification by American Joint Committee on Cancer (AJCC-UICC, 1997) [6]. Tumors of superficial nature classified as T1 or Ta while with deep muscular invasion were assigned as T2 or T3. Similarly tumor grading was done as G1 (low grade) and G2 or G3 (high grade). Patients were followed for recurrence (the number of times patient develops the tumor) every three months for 36 months with cytology and cystoscopy. The clinical and pathological characteristics of all the patients are summarized in Table 1.

**Table 1:** Clinical and pathological features of the patients diagnosed with bladder Carcinoma

**Case no.**	^@^**Age/ ^$^Gender**	**Stage**	**Grade**	**#Recurrence**	**MSI**
BC 1	45/M	T2	High, G3	2	*High (BAT – 26, BAT – 40)
BC 2	59/M	T1	High, G2	1	High (BAT – 26, BAT – 40, D9S1851)
BC 3	66/M	Ta	Low, G1	0	High (D9S283, D9S1851)
BC 4	39/M	T2	High, G3	0	High (BAT – 26, BAT – 40, D9S283)
BC 5	72/M	T2	High, G3	0	High (BAT – 40, D9S1851)
BC 6	59/M	T2	High, G2	1	Low (D9S1851)
BC 7	78/M	T2	High, G3	0	High (D9S283, D9S1851)
BC 8	52/M	Ta	High, G2	0	High (BAT – 40, D9S283, D9S1851)
BC 9	71/M	Ta	High, G2	0	**Low (BAT – 40)
BC 10	84/M	T1	High, G2	0	***MSS
BC 11	55/M	T1	High, G1	0	MSS
BC 12	53/M	T2	High, G2	0	Low (D18S58)
BC 13	52/M	T3	High, G3	0	High (D9S283, D9S1851)
BC 14	40/M	T3	High, G3	0	MSS
BC 15	55/M	T2	High, G3	0	MSS
BC 16	60/M	T2	High, G3	0	High (BAT – 40, D9S283, D18S58)
BC 17	66/M	T1	High, G3	1	High (BAT – 40, D2S123, D9S283, D9S1851, D18S58)
BC 18	80/M	T1	High, G3	1	High (BAT – 26, D9S283, D18S58)
BC 19	42/M	T1	High, G2	0	Low (D9S283)
BC 20	73/M	T3a	High, G3	0	MSS
BC 21	55/M	T2	High, G3	0	Low (D2S123)
BC 22	58/M	T2	High, G3	0	High (BAT – 26, D2S123)
BC 23	70/M	T1	High, G2	0	Low (BAT – 26)
BC 24	53/M	Ta	High, G3	0	High (D9S1851, D18S58)
BC 25	60/M	T2	High, G3	0	High (D9S283, D9S1851)
BC 26	54/M	Ta	Low, G1	0	MSS
BC 27	72/M	Ta	High, G2	0	MSS
BC 28	58/M	T1	High, G3	0	Low (D9S283)
BC 29	80/M	T2	High, G3	0	High (D9S283, D9S1851)
BC 30	64/M	Ta	High, G2	2	Low (BAT – 40)
BC 31	74/M	Ta	High, G3	0	High (D9S283, D9S1851)
BC 32	60/M	T1	Low, G1	0	MSS
BC 33	41/M	T1	Low, G1	3	Low (BAT – 26)
BC 34	66/ F	T2	High, G3	0	High (D2S123, D9S283)
BC 35	53/M	T1	Low, G1	0	Low (D2S123)
BC 36	66/M	T1	High, G2	1	High (D2S123, D9S283, D18S58)
BC 37	55/M	T2	High, G3	0	High (BAT – 26, BAT – 40, D9S1851)
BC 38	65/M	T2	High, G3	0	Low (D18S58)
BC 39	69/M	T1	Low, G1	0	Low (D9S283)
BC 40	74/M	T1	High, G2	1	MSS
BC 41	72/ F	T2	Low, G1	0	MSS
BC 42	71/M	T1	High, G3	1	Low (D9S283)
BC 43	64/M	T2	High, G3	0	High (BAT – 40, D9S1851)
BC 44	73/M	T1	High, G2	1	Low (D9S283)

^@^Age = (years); ^$^Gender = (M: Male/F: Female); *MSI – H = MSI – High; **MSI – L = MSI – Low; ***MSS = microsatellite stable

# Recurrence (0, 1, 2, 3) = Number of times the tumor recurred

### DNA isolation

Superficial tumor tissue specimens of histologically confirmed bladder tumors and peripheral blood (frozen) of the same patient were processed for DNA isolation using phenol-chloroform extraction method [7].

### MSI analysis

Table 2 demonstrates the characteristic features of the microsatellite markers evaluated in the present study. The primer sequences for mono and dinucleotide microsatellite markers were searched from human genomic database. Polymerase chain reaction (PCR) amplification of DNA was done using primers of concentration of 6 pmol, 200 μM dNTPs, 10 mM Tris – Cl (pH 8.3), 50 mM KCl, 1.5 mM MgCl_2_, 0.25 units of Taq polymerase (MSI, Fermentas), 100 ng DNA and 2 μCi [α-^32^P] dCTP (specific activity: 4000 Ci/mM) (BRIT, India) in a volume of 25 μl. PCR conditions involved an initial denaturation at 95°C for 3 min followed by 30 cycles (95°C for 1 min, 50°C to 60°C for 2 min and 72°C for 3 min) and a final extension at 72°C for 8 min. PCR products were mixed with equal volume of formamide loading dye (95% formamide, 20 mM EDTA, 0.05% bromophenol blue, 0.05% Xylene cyanol), denatured for 5 min at 95°C and loaded onto 8% polyacrylamide gel containing 7 M urea. Gels were run at 55 W for 2 hours, transferred onto a Whatman sheet followed by an exposure to X-ray film (Kodak) for desired time and then developed.

**Table 2:** Characteristic features of microsatellite markers examined in urinary bladder tumors

**Microsatellite marker**	**Repeat pattern**	**Chromosomal location**	**~ PCR product size**
BAT 26	(A)_26_	5^th ^intron of hMSH2, 2p	117 – 130 bp
BAT 40	(A)_40_	2^nd^intron of β hydroxy steroid dehydrogenase	94 – 112 bp
BAX (38 – 41)	(G)_8_	19q13.3 – q13.4	94 bp
TGFβ RII (665 – 737)	(A)_10_	3p22	73 bp
IGFIIR (4030 – 4140)	(G)_8_	6q26 – 27	110 bp
HMSH3 (381 – 383)	(A)_8_	5q	150 bp
D2S123	(CA)_13_TA	2p16	197 – 227 bp
	(CA)_15_(T/GA)_7_		
D9S283	(CA)n	9q13 – q22	178 – 203 bp
D9S1851	(CA)n	9q22.3	143 – 159 bp
D18S58	(GC)_5_GA(CA)_17_	18q22.3	144 – 160 bp

The tumor was designated unstable if its PCR product had altered band pattern when compared to alleles in corresponding matched blood DNA [8]. Out of he motifs studied, BAT-26 & BAT-40, the mononucleotide poly A repetitive loci have been shown to exhibit polymorphism [9]. The change either borderline or major deletions/ insertions at this loci is compared in tumor tissue of the same patient with the normal tissue in colorectal tumors [8]. Tumors were called MSI-High (MSI-H) when they showed instability at > 30% of loci and MSI-Low (MSI-L) if they showed at or less than 30% of loci.

### Statistical analysis

Statistical tests, including, 2 × 2 contingency table, Fisher's exact probability test (one or two tailed), Karl Pearson's correlation test were applied to assess the relation between the microsatellite instability in tumors and clinicopathological parameters. A student t test was applied to compare the number of genomic alterations between tumors of different grades and stages.

## Results

A panel of ten microsatellite markers situated on chromosomes 2, 3, 5, 6, 9, 18 and 19 were screened to look for microsatellite instability in superficial tumor tissues and compared with blood DNA. Alterations were detected in 32 of 44 patients (72.7%).

**Figure 1:**
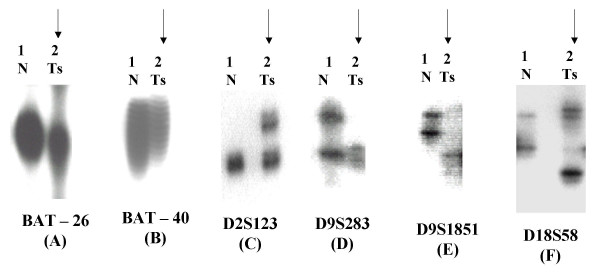
Changes in allelic pattern indicated by an arrow, observed in superficial tissue (Ts) as compared to blood (germline DNA, N) of patients with bladder carcinoma: (A) Deletion at BAT – 26; (B) Insertion at BAT – 40; (C) Insertion at D2S123; (D) Loss of heterozygosity at D9S283; (E) Biallelic alteration at D9S1851 and (F) Deletion at D18S58

**Figure 2:**
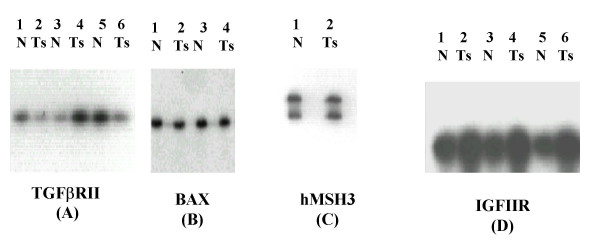
Superficial tumor tissues and blood (control) of bladder tumor patients demonstrated no change at (A) TGFβ RII; (B) BAX; (C) hMSH3 and (D) IGFIIR

Out of the six mononucleotide microsatellite markers analyzed, only BAT – 26 and BAT – 40 in 17.7% and 24.4% of the cases could demonstrate changes respectively while TGFβ RII, IGFIIR, hMSH3 and BAX were microsatellite stable (MSS). BAT-26 & BAT-40 exhibited borderline changes in tumor DNA as compared to control DNA. We also sequenced the tumor & normal PCR product for these microsatellites to prove the change. The dinucleotide markers- D2S123, D9S283, D9S1851 and D18S58 exhibited altered electrophoretic migration pattern in 15.5%, 40%, 31.1% and 17.8% bladder tumors respectively. The most frequent microsatellite alteration was detected on the markers of chromosome 9 (D9S283 followed by D9S1851) (Fig 1 and 2).

A significant association of MSI – H with T2 or T3 stage tumors (2 × 2 contingency table, p = 0.05) and ≥ G2 grade tumors (Fisher's exact probability test, two tailed, p = 0.08) was observed (Table 1).

Total genomic alterations were analyzed among different stages and grades. Changes were comparable among (24/44) T1-Ta and (20/44) T2-T3 tumors that included 35 (13 insertions, 6 deletions, 10 LOH and 6 BA) and 31 (10 insertions, 8 deletions & LOH and 5 BA) respectively (p > 0.05). High grade (G2-G3) (37/44) carcinomas encompassed 63 alterations (24 insertions, 14 deletions, 15 LOH and 10 BA) and low grade (G1) (7/44) bladder tumors had only 5 (1 each deletion & BA and 3 LOH) (p = 0.01)

Recurrence of tumors (mean duration of 36 months) was correlated with MSI in 24 superficial (Ta-T1) tumors in patients who turned up for regular follow up (Table 1). This group comprised 18 high grade and six low grade tumors. In the high grade tumors, 13 were MSI+, and 8 of them (61.5%). showed recurrence while only one (1/5, 20%) MSI- recurred. Amongst low grade tumors (6), 2 recurrences were noted only in MSI+ group and none of the MSI- showed recurrence (Fisher's exact probability two tailed test) (p = 0.02 for high grade tumors) and (p = 0.04 for low grade tumors).

## Discussion

This present work is a continuation of the previous published work where thirty bladder tumors were analyzed for the presence of MSI at BAT-26, BAT-40, TGFβ RII, IGFIIR, hMSH3 and BAX. The initial results encouraged examining the role of MSI/ LOH in more number of tumors with expanded panel of markers [7]. In this paper, 44 patients of bladder cancer are examined for MSI at BAT-26, BAT-40, TGFbRII, IGFIIR, BAX, & hMSH3 & D2S123, D9S283, D9S1851 & D18S58 dinucleotide repeat motifs. The MSI results are further analysed with clinicopathological features: stage and grade of the tumors & its recurrence in due course of time. The importance of MSI in the diagnosis of recurrence in superficial cases irrespective of grade & thus advocate the bladder cystectomy as a treatment modality in these cases.

Genomic instability measured by changes in tumor tissues as compared to blood of the same patient at repetitive loci was detected in 72.7% cases of bladder carcinoma. It differs from previous observation, which shows infrequent occurrence of MSI in TCC using different microsatellite markers [10]. However, low frequency of MSI with alterations of dinucleotide repeats in TCC of the urinary tract was found as 21% and 16.6% in two independent studies [11,12]. MSI and allelic loss in a series of 26 upper urinary tract tumors using 5 informative microsatellite markers were examined & this study supports the presence of MSI in upper urinary tract which is rare event in bladder cancer [13]. Another study describes MSI and loss of respective MMR protein by immunostaining in a patient with a urothelial carcinoma of the ureter and a strongly positive history of cancer, who was subsequently found to have HNPCC [14].

In the present study a significant association of MSI with tumor stage and grade in sporadic bladder tumors suggested MSI as an early event in tumorigenesis. These results confirm the previous finding where MSI examined in TCC of bladder with low stage and grade using few microsatellite markers mostly confined to chromosome 9 [15]. Another study reports 100% tumor instability as determined by dinucleotide repeat analysis in 14 cases of urinary bladder of different stages and grades [16]. Many studies show relatively high proportion of tumors with mutations in di, tri, and tetra nucleotide repeat motifs, although each tumor exhibits only few such mutations [4]. Recently, a novel form of MSI, termed as EMAST (elevated microsatellite instability at selected tetranucleotide repeats) has been found to be significantly associated with mutations in p53 among the bladder cancer tumors, but no indication of elevated EMAST in tumors with abnormal p53 staining without mutation. EMAST likely reflects a particular pattern of somatic events that are interactive with p53 mutation, particularly common in skin cancer and limited to non-invasive disease in bladder cancer [17]. The difference between these studies and ours may be attributed to the number and identity of microsatellite motifs studied.

Despite clear-cut prognostic differences, genetic alterations were comparable in superficial (Ta-T1) and invasive bladder carcinomas (T2-T3) suggesting the role of MSI in progression of bladder cancer as well. However, strong association of MSI – H with T2-T3 and G2-G3 was observed. MSI at ≥ 30% of loci has been found in 59.4% (19/32) of TCC bladder, which is not in accordance with reported earlier [11,12]. A good association of MSI – H with high grade superficial tumors may help in deciding radical surgery to begin with.

Bladder cancer presents as superficial tumor in 75% of the patients, which can easily be removed by transurethral resection (TUR). Around 60–80 % of these treated patients develop recurrence in due course of time. Out of them, 15% progress to higher grade and stage. With so much potential for recurrence, patients need to be followed up with cystoscopy at regular intervals. Although many new tumor markers have been proposed but all have limitations with respect to execution and interpretation in predicting the recurrence of bladder tumors [18]. Among the molecular markers, alterations in p53, p21^WAF/C1P1^, Rb, c-erb B-2 are reported to be associated with tumor recurrence and progression but little is known to address MSI [19]. MSI analysis gives higher sensitivity and easy to execute among other molecular markers, thus making it a valuable marker for detection of recurrence. To the best of our knowledge, this is the first study reporting MSI as a good prognostic marker that correlates with risk of recurrence in superficial (Ta-T1) tumors irrespective of the grade. This may help in deciding radical treatment at an early stage. We could not study the genetic changes during the progression of tumor, which means the extension of superficial tumor confined to the mucosa and submucosa to deep musculature of the bladder. Limitation of this study is a small number of patients but initial trends show a strong correlation of MSI with recurrence irrespective of the grade of the tumor. Further multicentre trial is needed to prove this concept.

## Conclusions

MSI has been observed to play important role in evolution, initiation and progression in bladder tumors. Patients with high grade superficial disease are reported to have higher incidence of MSI. Also high frequency of MSI in superficial tumors showing recurrence irrespective of grade may provide an indication for more radical approach to improve the survival.

## Competing interest

The author(s) declare that they have no competing interests.

## Authors' contribution

MV carried out the molecular genetic studies, participated in analyzing the data & drafted the manuscript. AM provided the clinical material & information, helped in analyzing the data & designing the manuscript. RDM helped in manuscript drafting. BM participated in its designing of the study & manuscript. All authors read and approved the final manuscript.

## Pre-publication history

The pre-publication history for this paper can be accessed here:


